# Cancer neoepitopes viewed through negative selection and peripheral tolerance: a new path to cancer vaccines

**DOI:** 10.1172/JCI176740

**Published:** 2024-03-01

**Authors:** Pramod K. Srivastava

**Affiliations:** Department of Immunology and Carole and Ray Neag Comprehensive Cancer Center, University of Connecticut School of Medicine, Farmington, Connecticut, USA.

## Abstract

A proportion of somatic mutations in tumors create neoepitopes that can prime T cell responses that target the MHC I–neoepitope complexes on tumor cells, mediating tumor control or rejection. Despite the compelling centrality of neoepitopes to cancer immunity, we know remarkably little about what constitutes a neoepitope that can mediate tumor control in vivo and what distinguishes such a neoepitope from the vast majority of similar candidate neoepitopes that are inefficacious in vivo. Studies in mice as well as clinical trials have begun to reveal the unexpected paradoxes in this area. Because cancer neoepitopes straddle that ambiguous ground between self and non-self, some rules that are fundamental to immunology of frankly non-self antigens, such as viral or model antigens, do not appear to apply to neoepitopes. Because neoepitopes are so similar to self-epitopes, with only small changes that render them non-self, immune response to them is regulated at least partially the way immune response to self is regulated. Therefore, neoepitopes are viewed and understood here through the clarifying lens of negative thymic selection. Here, the emergent questions in the biology and clinical applications of neoepitopes are discussed critically and a mechanistic and testable framework that explains the complexity and translational potential of these wonderful antigens is proposed.

## Introduction

Revolutionary advancements in high-throughput DNA-sequencing approaches led to the identification of numerous tumor-specific mutations, some of which generate new epitopes (neoepitopes). Tremendous effort has gone into leveraging these neoepitopes toward development of cancer vaccines and other immunotherapies. However, the overwhelming majority of neoepitopes do not control tumors in vivo, necessitating a carefully informed framework for selection of optimal neoantigens for therapeutic development.

*“I would not give a fig for the simplicity this side of complexity, but I would give my life for the simplicity on the other side of complexity.”*

This quote, attributed to both the American physician and poet Oliver Wendell Holmes Sr. and his son, the US Supreme Court justice Oliver Wendell Holmes Jr., aptly describes scientific pursuit of understanding in the wake of a new discovery. As I will discuss here, after initial enthusiasm over the discovery of cancer-specific epitopes was stymied by preclinical and clinical failures, ongoing studies revealed a set of principles that may better explain the biology of cancer neoepitopes and better identify neoepitopes with therapeutic utility. Considering that simplicity on the other side of complexity derives from the pursuit of simplicity this side of complexity, I would give bushels of gold for the simplicity this side of complexity as well.

The search for what makes cancers different from normal tissues makes for a long and winding tale. Cancers are the cause of (often) fatal disease, and they generally look different from normal tissues under a microscope. And yet, what makes them different, structurally speaking, has been unclear. The fact that the immune system detects cancers and can eliminate them has been known for some time (1), and since the immune system generally attacks only that which is different from normal, the thought that cancers harbor some entity that is truly different has long been a focus of attention of immunologists. Enter cancer neoepitopes.

Cancer neoepitopes are altered peptides that are presented by the MHC molecules of cancer cells or antigen-presenting cells (APCs). The alterations typically derive from missense point mutations in cancer cells; however, epitopes derived from introns (2), antisense transcripts (3), untranslated regions of genes (4), other noncoding regions of the genome (5), or insertions/deletions are also neoepitopes, if they are specific to cancers.

## Simplicity before the complexity: 

## foundational neoepitope discoveries

**Figure 1:**
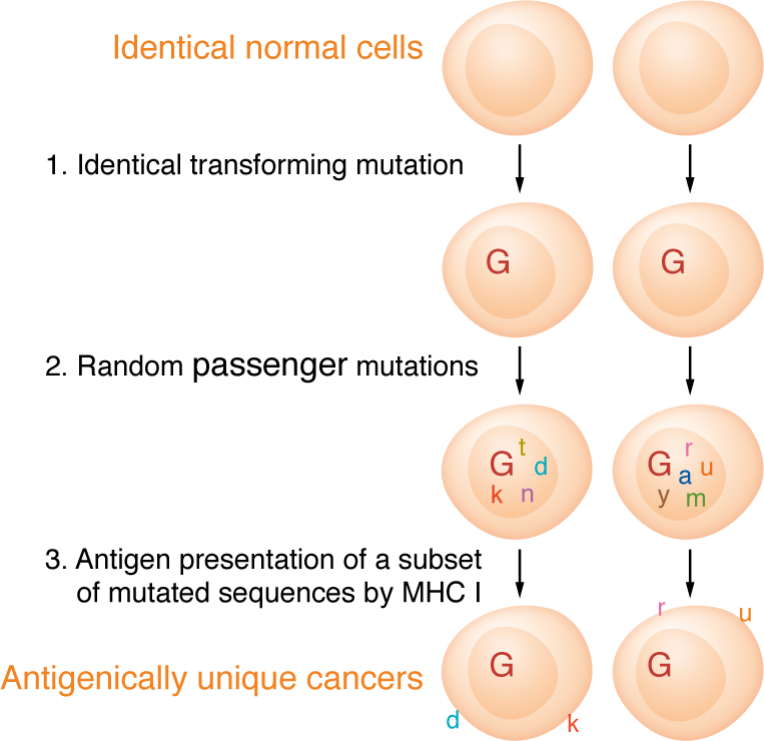
Model to explain the individually distinct antigenicities of cancers. Identical normal cells are transformed by a driver mutation G, followed by multiple cell divisions (not shown) of each cell, resulting in random (and therefore individually distinct) mutations in two otherwise-identical cancer cells. A small proportion of these mutations are able to be presented by MHC molecules, resulting in individually distinct neoepitopes and immunopeptidomes. Adapted with permission from *Advances in Cancer Research* (6).

The existence of natural cancer neoepitopes was first hypothesized in 1993 (6), although the idea may have been implicit in earlier work (7–9). Neoepitopes were hypothesized to explain the extraordinary observation in mouse and rat tumors that each individual tumor is antigenically unique (ref. 1, see ref. 10 for review); it was suggested that random passenger mutations (which arise with each cycle of DNA replication as a result of less than complete fidelity of DNA polymerases) occasionally result in peptide sequences that could be presented by the host MHC and recognized by the immune system as foreign (Figure 1). The vast size of the genome, as compared with the rarity of random mutations, would make it highly unlikely that the same random mutation could occur twice, explaining the uniqueness of antigenicity of individual tumors (1, 10). Since driver mutations would be common to many cancers, they were specifically excluded as being sources of cancer neoepitopes in that early hypothesis.

The hypothesized neoepitopes and the current excitement about them are best viewed in the context of the zeitgeist of cancer immunology of the 1990s. Boon and colleagues had developed an elegant method to identify the antigens recognized by cytotoxic CD8^+^ T cells and had used it to define the epitopes of CD8^+^ T cells (7). These epitopes turned out to have normal sequences and were not cancer specific; yet, the observation launched a new phase of cancer immunology on steroids. Although lone voices drew attention to the fact that there was nothing cancer specific about these epitopes (11), it took over two decades and two failed large, randomized studies in melanomas (12) and lung cancers (13) for the enthusiasm for these antigens as cancer vaccines to subside.

The high-throughput DNA-sequencing approaches that would allow us to sequence normal and cancer DNA from the same patient or mouse matured in early 2000s and promised to finally identify cancer-specific antigens. A small number of studies with murine cancers (14–18) had already identified the epitopes recognized by CD8^+^ and CD4^+^ T cells that were mutated in cancers and were thus cancer specific (Table 1); indeed, one could immunize mice with these peptides and elicit tumor rejection. Such mutated neoepitopes were also shown to be present in human cancers (19–21); of course, immunizations of patients to elicit protection from human cancers were not being tested at this time, although several studies had noticed a correlation between the presence of mutated neoepitopes and a favorable clinical course (22, 23). The premise for the excitement over identifying cancer-specific mutations using DNA sequencing was that it would eliminate the cumbersome step of having to generate cancer-specific CD8^+^ or CD4^+^ T cells to get to the neoepitopes. Comparison of normal and cancer sequences would be enough to get us to the cancer-specific neoepitopes in this brave new world. By predicting a number of potential MHC-binding neoepitopes in human breast and colorectal cancers, Allison and colleagues provided a first glimpse of the power of these new approaches (24).

Castle et al. first used DNA sequencing to identify several hundred nonsynonymous tumor-specific mutations in B16 mouse melanoma and neoepitopes derived from them (25). They tested two predicted neoepitopes in tumor rejection assays and observed both to provide significant tumor control. Three papers followed in quick succession (26–28), and using different tumor models as well as some individual variations, each conformed to the basic outline of Castle et al. Cancer-specific mutations could be identified and the resulting neoepitopes could be used to protect from tumor growth in vivo. Consistent with a role of neoepitopes in the natural immunogenicity of tumors, early studies showed that tumors arising in immune-compromised mice have a distinct and highly immunogenic neoepitope profile (29, 30). Even in immune-competent mice, the presence or absence of neoepitopes appeared to alter the course of natural tumor growth (31).

Among neoepitopes, the focus has mostly been on MHC I–presented antigens, largely because most tumors do not express MHC II and because MHC II–restricted antigens are more difficult to predict (32). Ironically, MHC II–restricted neoepitopes, which elicited tumor control of MHC II–deficient tumors, were among the very first neoepitopes to be identified (14, 18) 20–25 years ago. MHC II–presented neoepitopes were also identified using the genomics approach (refs. 33, 34; Table 2). Alspach et al. (34) identified an MHC II–presented neoepitope of the T3 sarcoma but did not report immunization of mice with it. They showed that “CD4^+^ T cell responses are required for optimal priming of MHC-I restricted CD8^+^ T cells and their maturation into CTL [cytotoxic T lymphocytes]”. They further observed that expression of the neoantigen that harbors the MHC II–presented neoepitope is required in the tumor microenvironment for the CD4 response to be effective. An additional MHC II–presented neoepitope, and its ability to facilitate tumor control by coimmunization with an MHC I–presented neoepitope, has been reported more recently (35, 36).

MHC I–neoepitope interactions have also been examined through structural approaches. The visualization of peptide-MHC complexes (37, 38) and, later, their complexes with T cell receptors (39, 40) transformed immunology. A pair of analyses used the collective wisdom of such studies to identify features associated with immunogenicity (41, 42). In hindsight, it is not surprising that these features — summarized as enrichment in hydrophobic and aromatic amino acids — are enriched in immunogenic epitopes, as they reflect the basic physical principles that govern protein-protein recognition (43). Neoepitopes abide by the same physical principles as other protein structures and, as a class, do not “look” structurally different or unusual (44–46).

Radiation of cancers is one of the key modalities of cancer treatment, and radiation is, of course, an established mutagen. Hence, the possibility that some of the mutations caused by radiation elicit immune response that may be able to control cancers in vivo has been explored. Demaria and colleagues demonstrated the ability of neoepitopes of a mouse model of triple-negative breast cancer to enhance the therapeutic efficacy of radiation alone (47). More recently, Schreiber and colleagues showed that radiation of nonimmunogenic tumor cells with a very low mutational burden can induce neoepitopes that can elicit potent T cell response and tumor rejection as well as sensitivity to checkpoint blockade (48). These observations are reminiscent of the chemical mutagen-induced highly immunogenic tum- mutations in nonimmunogenic tumors (8, 9).

Collectively, these observations helped anchor the neoepitopes firmly in a mechanistic framework, which provided the foundation of the emergent broader complexity.

## The complexity: mechanistic expectations versus reality

Tables 1 and 2 show lists of neoepitopes that have been shown to mediate or facilitate tumor control in vivo. The earliest neoepitopes (Table 1) were identified as those recognized by tumor-specific CD4^+^ or CD8^+^ T cells. Neoepitopes that elicit tumor control in vivo and were identified by high-throughput DNA sequencing are shown in Table 2. A small number of other cancer epitope databases exist (49); their scope is different from that of Tables 1 and 2, which are restricted to neoepitopes that have been tested for their ability to mediate tumor control in vivo. Currently completed clinical studies are at too early a stage to provide definitive evidence that neoepitopes mediate tumor control in vivo in humans and, hence, are excluded from these tables. Studies that describe mixtures of neoepitopes that mediate tumor control in vivo, but where it is not possible to ascribe the activity to one or more identified neoepitopes are also generally not included in Tables 1 and 2. Some studies with mixtures of neoepitopes show impressive tumor control in vivo and in mouse models that are similar to actual clinical scenarios (50). Most importantly for our present purposes, scrutiny of the properties of the tumor-controlling neoepitopes in Tables 1 and 2 illustrates the challenging complexity of neoepitope biology, as discussed below.

### MHC I–peptide affinity is not predictive of antitumor activity in vivo in mice or humans.

How do we go from identifying mutations to predicting neoepitopes? In a landmark paper, Sette and colleagues (51) analyzed “the relationship between binding affinity for HLA class I molecules and immunogenicity of discrete peptide epitopes” with respect to viral antigens. They “found that an affinity threshold of approximately 500 nM (preferably 50 nM or less) apparently determines the capacity of a peptide epitope to elicit a CTL response.” This foundational observation has solidly sustained our understanding of what is an epitope in hundreds of studies with viral and model antigens. All studies predicting neoepitopes from mutations in cancer have used this algorithm to discriminate between the few mutations that are likely to be presented and be immunogenic over those that are not (Figure 1) (25, 27, 28). A surprisingly large number of algorithms for predicting cancer neoepitopes have now been published (52–60); almost all algorithms share the critical principle that the neoepitopes must bind the relevant MHC I alleles with high affinity.

Duan et al. (26) performed broadly the same study as previous (25) and later efforts (27, 28) and came to different conclusions. Exome sequences of two mouse fibrosarcomas were compared with normal counterparts, and the cancer-specific mutations were used to predict high-affinity neoepitopes; surprisingly, none of the predicted neoepitopes elicited tumor control. Soon after, Martin et al. (61) performed an essentially similar study with a mouse model of ovarian cancer. They predicted a number of neoepitopes that bind MHC I with high affinity, but none of the 17 predicted neoepitopes elicited tumor control in vivo.

Duan et al. (26) reasoned that except for single amino acid substitutions, neoepitopes are identical to the unmutated sequences, and hence the mechanisms of peripheral tolerance may also inhibit the response to neoepitopes. They argued that it may therefore be useful to select peptides by difference-from-self rather than high affinity for MHC I. To quantitate difference-from-self, they developed the simple algorithm of subtracting (or dividing) the MHC I–binding affinity of the unmutated sequence (or some derivative of this number) from the MHC I–binding affinity of the mutated neoepitope and named it the differential agretopic index (DAI). Ranking neoepitopes by DAI led to enrichment of peptides that elicited tumor control in both tumors. Strangely, all the tumor-controlling neoepitopes turned out to have poor affinity for any MHC I allele. These affinities were so low (i.e., the IC_50_ values so high), that these neoepitopes would have been eliminated from consideration by the Sette algorithm. In spite of such low affinities, the tumor control elicited by these low affinity MHC I–binders was dependent on CD8^+^ T cells in vivo.

In light of these inconsistencies, Brennick and George et al. (62) performed an unbiased analysis of the tumor control activity of every mutated peptide in the exome of the MC38-FABF tumor (a chemically induced murine tumor) without making any predictions. At the time of this writing, this is the only unbiased study that looks for neoepitopes that mediate antitumor efficacy in vivo. Other unbiased studies have looked for CD8 immunogenicity, without examining efficacy (63–67). Since the endpoints of Brennick and George et al. (i.e., antitumor efficacy in vivo) are different from those of other unbiased studies (CD8^+^ T cell immunogenicity in vitro), the results and conclusions are also different and reveal an important complexity: immunogenicity and efficacy are not the same and should not be conflated.

**Figure 2:**
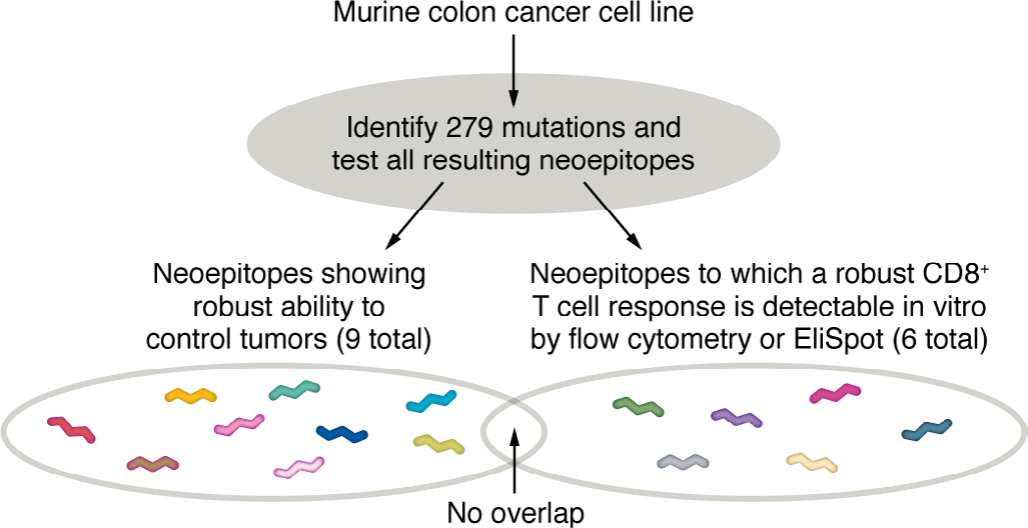
Dissonance between tumor control and CD8^+^ T cell response, as measured in vitro as well as pMHC I affinity. Summary of the outcomes of an unbiased analysis of tumor control and CD8^+^ T cell responses elicited by 279 neoantigens isolated from a murine cancer cell line (62). The candidate neoepitopes that elicited tumor control in vivo did not elicit CD8^+^ T cell response, as measured by flow cytometry, even as their activity in vivo was CD8 dependent, as shown by depleting CD8^+^ T cells in vivo. The candidate neoepitopes that did elicit CD8^+^ T cell responses by flow cytometry did not elicit tumor control in vivo. Only 1 of 9 neoepitopes that mediated tumor control in vivo had a high affinity to MHC I (not shown).

Brennick and George et al. identified nine neoepitopes (of 279 candidates) that were effective in controlling tumor growth (Figure 2). Eight of these nine neoepitopes had very low affinities for MHC I (between <3,000 and >30,000 nM IC_50_), while one had a high binding affinity. This unbiased analysis suggests that approximately 4% of the mutations in this tumor yield tumor controlling neoepitopes. This is a larger proportion than conventionally estimated, because it does not eliminate peptides because of poor affinity for MHC I (Table 2 and Figure 2). It also shows that the Sette algorithm does not appear to apply to cancer neoepitopes as it does to epitopes derived from viral antigens. It should be noted that Sette et al. presciently noted 20 years ago that “It is also possible that self-derived antigenic peptides may be characterized by relatively low MHC binding affinity because of selective elimination by thymic education and/or T cell tolerance of T cells reactive against high affinity MHC binding peptides” (51). That caveat has now come fully alive in cancer neoepitopes.

Ghorani et al. (68) and Rech et al. (69) tested these ideas in the human setting. Using data from tumor and normal exome sequences of patients with melanoma and lung cancer who had received checkpoint blockade, Ghorani et al. looked for correlations between genomic characteristics (mutational load, neoantigen load, mean DAI) and clinical outcomes (overall survival) as well as immune infiltration of tumors. They concluded that “the association between mean DAI, survival, and measures of immune activity support the hypothesis that DAI is a determinant of cancer peptide immunogenicity.” In a broadly similar but independent analysis in several thousand patients with all major cancers, Rech et al. concluded that the presence of neoepitopes with a positive DAI correlated with intratumoral T cell responses and that such neoepitopes “were also strong predictors of patient survival across tumor types.” Other studies have independently validated the contribution of a higher predicted binding affinity of a mutant peptide relative to the corresponding wild-type peptide in immunogenicity (70–73).

The observation that peptides that bind MHC I poorly (e.g., IC_50_ values of 30,000 nM) elicit effective CD8 responses runs contrary to the well-established paradigm and requires explanation. While competitive peptide binding (which generates the IC_50_ values) is the norm in measuring MHC I–peptide binding, other methods of binding were tested. Binding of peptides to RMA-S cells is one such method. These cells harbor a mutated transporter associated with antigen processing-2 (TAP2) such that peptides generated in the cytosol by proteasomal degradation are poorly transported from the cytosol to the endoplasmic reticulum, leading to poor loading of MHC I with peptides (74). Such “empty” MHC I molecules are unstable at 37°C but can be salvaged if an exogenous peptide is able to bind the “empty” MHC I molecules. Peptide binding by RMA-S cells thus converts the RMA-S cells from MHC I–negative into MHC I–expressing cells (75). Brennick and George et al. (62) used this assay to demonstrate the binding of neoepitopes to K^b^ or D^b^ with affinities as low as IC_50_ values of approximately 30,000 nM. The low-affinity peptides could also be eluted from the MHC I molecules and detected by mass spectrometry (MS) (31, 62).

These observations show that a low-affinity peptide–MHC I binding is still effective and leads to fruitful engagement of CD8^+^ T cells, which control tumor growth in vivo. In light of the observation that a single MHC I–peptide molecule on a target cell can elicit a CTL response (76), the functional significance of presentation of low-affinity peptides by MHC I is vastly underestimated. The antitumor activity of low-affinity MHC I–binding neoepitopes has now been reported in multiple tumors and from several independent laboratories (26, 31, 36, 62, 77).

### CD8 response measured in vitro is not predictive of antitumor activity in vivo.

Nearly all studies measure CD8^+^ T cell response to tumors, since CD8 response is indisputably essential for tumor control. In several studies with neoepitopes, a CD8 response, as measured by ELISPOT or flow cytometry, is indeed associated with tumor control in vivo (25, 27, 28), supporting the idea that the CD8^+^ T responses, as measured by ELISPOT or flow cytometry, are surrogates of antitumor efficacy in vivo. This idea persists in spite of the observation in mice (78–80) and in humans (81–83) that there is often little correlation between measurable CD8^+^ T cell responses and antitumor clinical activity. In fact, many studies with neoepitopes are limited to predicting or detecting CD8 responses and considering such response as proof of antitumor activity (84–89).

Three studies exemplify the disconnect between antitumor efficacy in vivo and CD8^+^ T cell immunogenicity. In a mouse model of ovarian cancer, Martin et al. identified 17 predicted neoepitopes with high affinity for MHC I (61). None of the 17 elicited tumor control in vivo, even as 7 of 17 elicited specific CD8 and/or CD4 responses. Vormehr et al. (89) reported that CD8^+^ T cells respond to a well-defined cancer neoepitope of the CT26 colon carcinoma are functionally irrelevant in vivo, i.e., do not elicit any tumor control despite strong immune response. Brennick and George et al. (62), who performed the unbiased analysis of all 279 mutation-generated candidate neoepitopes for their ability to mediate tumor control, observed, remarkably, that none of the nine neoepitopes that mediated tumor control elicited a measurable CD8^+^ T cell response in vitro, even though the tumor control mediated by them was CD8 dependent, as shown by in vivo depletion. Even more remarkably, several of the 279 mutant peptides tested did elicit a strong CD8^+^ T cell response, but these did not elicit tumor control (Figure 2)! If the mutations had been screened by CD8 immunogenicity, 8 of the 9 true positives would have been identified, and all of the “positive” candidates identified would have been false positives.

Viborg et al. (90) reported the activity of a DNA vaccine consisting of five neoepitopes of the mouse colon carcinoma cell line, CT26. The vaccine was highly effective in prophylaxis assays and also elicited CD8 response, as measured by tetramers. Upon deconvolution of the five neoepitopes, the antitumor activity was seen to reside only in neoepitopes 3–5, even as these neoepitopes elicited no CD8 response. Conversely, the CD8 response was elicited by neoepitopes 1–2, which elicited little tumor control!

### Presentation of a neoepitope by MHC I is not predictive of antitumor efficacy in vivo.

Several studies have identified MS-defined neoepitopes but looked for correlations with CD8 responses rather than tumor control in vivo, in mice or humans (91, 92). A single study has characterized the MS-defined neoepitopes for their ability to mediate tumor control in vivo and observed that, even among neoepitopes that are clearly presented by MHC I and which are of high affinity to MHC I, only a proportion (albeit a high proportion, 3 of 7) elicit tumor control in vivo (93). There are no obvious differences between the MS-defined neoepitopes that mediate tumor control from those that do not, further highlighting the fact that presentation of a neoepitope is no guarantee of efficacy.

### The clinical experience with neoepitopes is still in early stages.

The first clinical studies with neoepitopes were rooted in the observation of hsp70 and hsp90 chaperone peptides that were generated as a result of proteasomal degradation (94–97). These peptides were derived from self-proteins but also included viral epitopes or cancer neoepitopes (4, 91, 94). Thus, patients were immunized with HSP-peptide complexes isolated from autologous tumors. Despite promising activity in early trials (98, 99), the HSP-peptide complexes, administered as monotherapy, failed to show clinical activity in a phase III trial in patients with renal cell carcinoma (100). Although patients with early-stage disease benefited significantly, the intent-to-treat population as a whole did not. The approach continues to be pursued today with better tools of immunization and monitoring (101–103). In the high-throughput DNA-sequencing era (2012 and onward), patients with melanoma (104–106), glioblastoma (107, 108), pancreatic cancer (109), lung cancer (110), or bladder cancer (106) have been immunized with long peptides (or RNA encoding such peptides) containing neoepitopes that were predicted to be presented by MHC I. In some studies, such vaccines were combined with PD1 blockade (106, 111, 112). All studies showed safety, feasibility, and immunogenicity; however, the immune responses observed have not been consistent with the proposed mechanism of action for the vaccine. For example, in several studies although the immunizing neoepitopes were predicted to be presented by MHC I (and some were shown to be so presented), the predominant immune response was a CD4 response, although weaker CD8 responses were also detected (105, 108, 111, 113). In an exploratory analysis, Rojas et al. (109) linked T cell expansion after vaccination (interpreted as response to vaccine) with improved outcomes among patients with pancreatic cancer.

Early-phase clinical trials are not meant to show proof of clinical activity. In some studies, the clinical activity observed was similar to what one would expect from checkpoint blockade alone; in others, the number of patients was too small to allow interpretation. The recent KEYNOTE-942 trial was the only study in which the patients were randomized between vaccination plus checkpoint blockade and checkpoint blockade alone. This trial, reported by the sponsors as a positive study (114), consisted of 157 patients with stage III/IV surgically resected melanoma who received vaccination with an mRNA neoantigen vaccine plus checkpoint blockade (*n =* 107) or checkpoint blockade alone (*n =* 50). The endpoint of relapse-free survival was tested in a one-way analysis and was significant; however, if a two-way comparison was made, this endpoint was not significant (114). Distant metastases-free survival was also significantly different in a one-way comparison.

Thus, the clinical experience with the neoepitope vaccines has been similar to cancer vaccine approaches in the past and has not been particularly revealing, clinically or mechanistically. It also has highlighted the dissonance between efficacy and T cell immunogenicity even more effectively than past studies.

Altogether, the complexity, as outlined here, runs counter to some key mechanistic expectations of T cell immunology. The remainder of this Review attempts to synthesize and reconcile the contradictions in the complexity.

## Simplicity on the other side of complexity

### A simplifying proposition.

**Figure 3:**
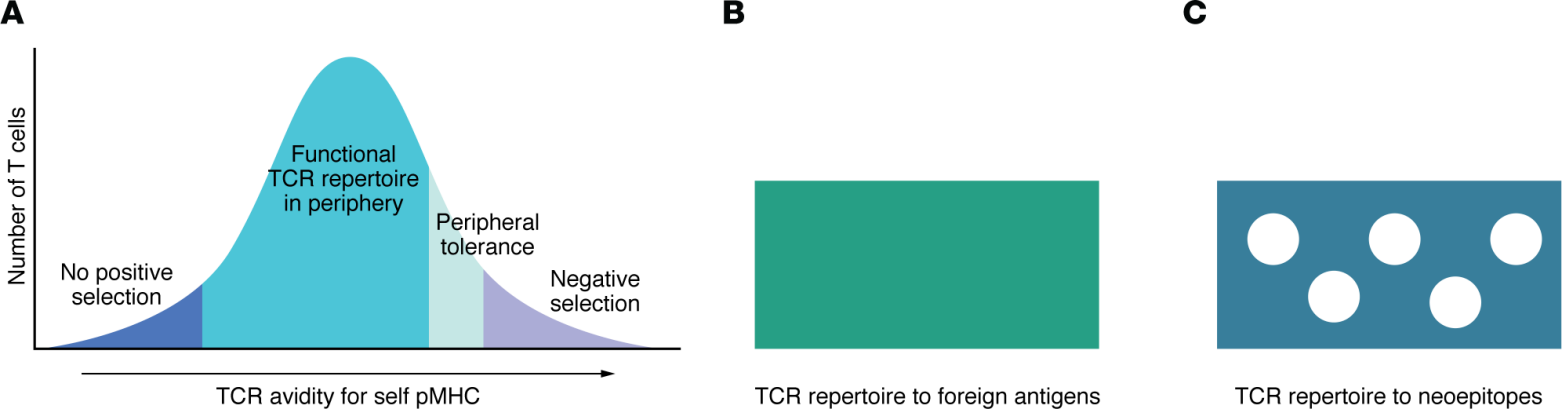
A view of the key differences between TCR repertoires against non-self (viruses and model antigens) and self (cancer neoepitopes). (**A**) Our standard understanding of positive and negative selection. (**B**) The TCR repertoire against non-self viral or model antigens is mostly untrimmed or unsculpted, because, with rare exceptions (as in ref. 119), these antigens have no self-counterparts. (**C**) In contrast, the TCR repertoire against cancer neoepitopes is profoundly trimmed or sculpted because of the self-evident similarity between cancer neoepitopes and self-antigens and the inherent cross-reactivity between the two. The TCR repertoire against cancer neoepitopes is thus proposed to be significantly narrower, qualitatively and quantitatively.

The knowledge of identity of neoepitopes that mediate tumor control in vivo versus those that do not has done little to explain why some neoepitopes are active in vivo and others are not. As discussed, their affinities for MHC, structures, and ability to elicit measurable CD8 responses do not shed light on this central question. It is posited here that the road to simplicity, i.e., reconciling the difficult complexities outlined in the previous section, lies in looking at the problem from the T cell end and, specifically, through the prism of negative selection (Figure 3).

In negative selection, a quintessentially Darwinian phenomenon, a vast proportion of T cells that have been positively selected are “screened” for avidity to self-antigens as presented on the medullary thymic epithelial cells. Cells expressing T cell receptors (TCRs) with the highest avidity for self-antigens are negatively selected (killed), while the remainder form the functional T cell repertoire of the individual (Figure 3A). TCRs that recognize foreign antigens, including model antigens (such as OVA, β-galactosidase) and viral antigens (influenza, lymphocytic choriomeningitis virus, hepatitis, herpes viruses, SARS-CoV-2 etc.), generally have no cross-reactivity with self-antigens, and hence the TCR repertoires to these antigens are broad, encompassing low, medium, and high avidity (115–118) and entirely unencumbered by any relationship with the self-antigens (Figure 3B). In very rare cases in which foreign antigens do cross-react with self-antigens, the corresponding TCRs are deleted (119). Here, the neoepitopes occupy a unique and interesting transitional ground. They are not foreign antigens and many differ by only one amino acid from the self-antigens that drive negative selection. This last point is reinforced by structural modeling of neoepitopes compared with wild-type counterparts, which suggests that, in many cases, single mutation in a neoepitope may have little effect on the overall conformation of the peptide in the binding groove (31, 46, 62, 93).

How does the negative selection process work with respect to neoepitopes (Figure 3C)? A large proportion of TCRs, particularly those with higher avidity, which recognize neoepitopes must also recognize the unmutated self, and the T cells that bear such TCRs must be deleted, leaving the anti-neoepitope T cell repertoire to have a low-avidity bias. Even among the T cells with TCRs with a low specific avidity for neoepitopes, those that approach the thresholds for thymic deletion or peripheral tolerance must be similarly eliminated or tolerized. Thus, the peripheral neoepitope-reactive T cell repertoire must be substantially restricted compared with the corresponding repertoire against foreign antigens, which is far less likely to be trimmed by negative selection (Figure 3C). TCR repertoire against neoepitopes is thus proposed to have a Swiss cheese quality by virtue of having many holes in the repertoire. Needless to say, this principle would not apply to TCRs that recognize neoepitopes derived from frameshift mutations, long noncoding RNA, or proteomically altered neoepitopes.

**Figure 4:**
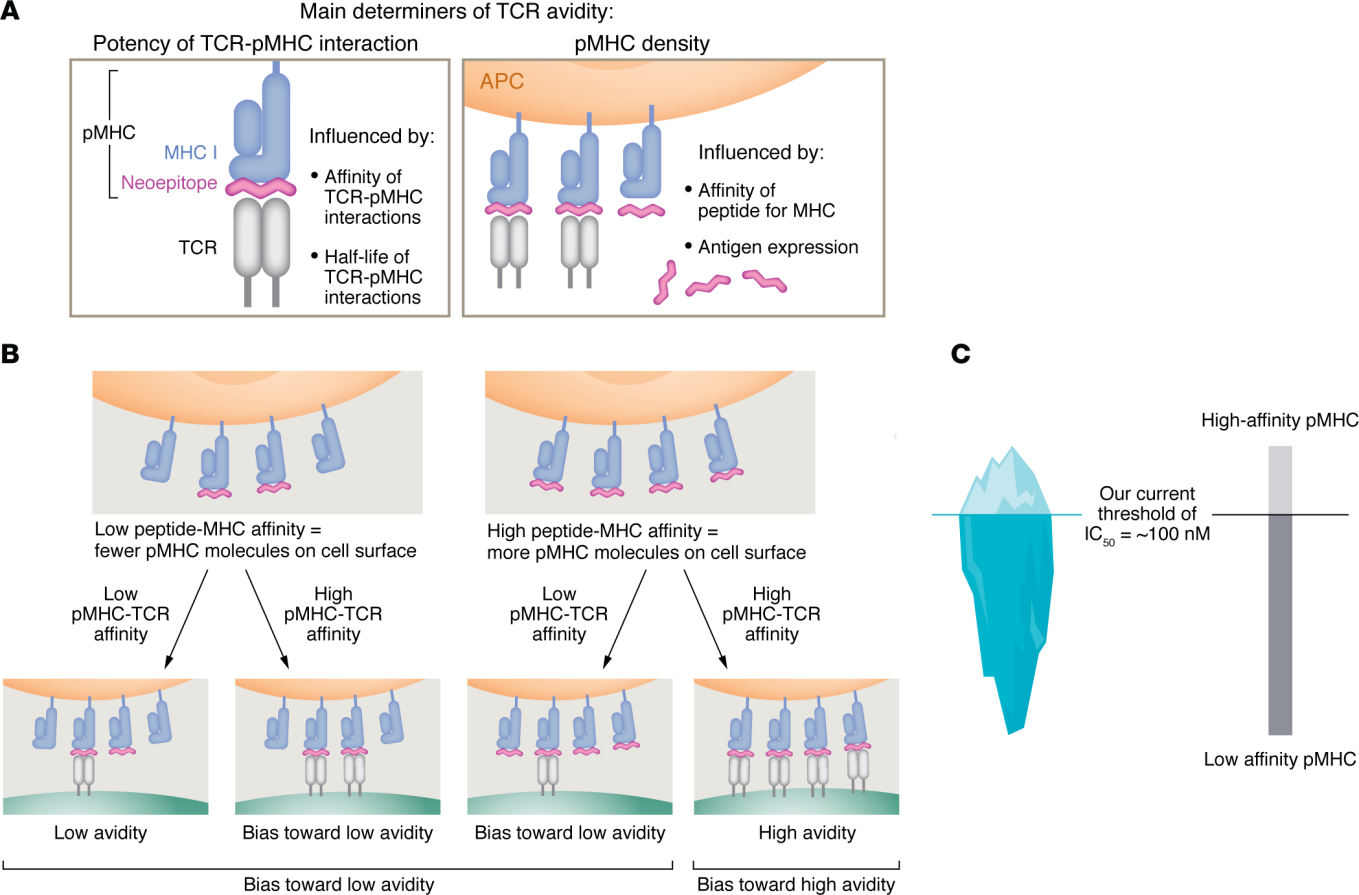
A unifying hypothesis: the simplicity on the other side of complexity. (**A**) The number of specific pMHC I complexes presented on the cell surface and pMHC-TCR affinity are the two major variables influencing TCR avidity. Other important variables, such as the level of expression of CD8α on T cells and the duration of the T cell–presenting cell interactions, are not shown. Adapted with permission from *Journal of Immunology* (120). (**B**) Other variables being the same, pMHC I affinity influences pMHC I–TCR avidity. If the pMHC I affinity is low and the pMHC-TCR affinity is low, the pMHC-TCR avidity is bound to be low. In case of a low pMHC affinity and a high pMHC-TCR affinity, the smaller number of pMHC molecules shall still drive the overall avidity to be biased toward the lower end. In case of a high pMHC affinity and a low pMHC-TCR affinity, the smaller number of pMHC molecules shall drive the overall avidity to be biased toward the lower end. If the pMHC I affinity is high and the pMHC-TCR affinity is also high, the pMHC-TCR avidity is bound to be high. Thus, in general, a low pMHC I affinity is most likely to drive the TCR avidity toward the lower end of the spectrum. (**C**) A metaphorical view of the universe of neoepitopes as an iceberg. The tip of the iceberg (the smaller component) harbors the high-affinity pMHC ligands while the submerged portion (the larger component) harbors the more abundant low-affinity pMHC ligands. The water level depicts the currently accepted threshold of productive pMHC affinity corresponding to an IC_50_ of about 100 nM.

Several components of peptide MHC–TCR (pMHC-TCR) interactions contribute to overall T cell avidity. These include pMHC-TCR affinity, number of TCR molecules on the T cells, and the number of pMHC molecules on the APCs (120) (Figure 4A). The first two are the attributes of T cells, while the third is an attribute of the APC. It is in examining the third attribute that some key aspects of complexity can be resolved (Figure 4B). A higher pMHC affinity accompanied by higher abundance of expressed peptides (i.e., the antigens themselves) will lead to a higher number of pMHCs, which will contribute to a higher overall T cell avidity. Conversely, a lower pMHC affinity and poor expression of antigen (as in lower transcripts per million) will contribute to a lower T cell avidity. It is posited here that it is for this reason that the universe of effective cancer neoepitopes is biased toward neoepitopes that have a poor affinity for MHC I and that are not abundantly expressed (Table 2).

It is instructive to consider the role of pMHC-TCR affinity in avidity (Figure 4). For the low pMHC affinity peptides, two scenarios can be envisaged (Figure 4B). If the pMHC-TCR affinity is low, both variables would be low and the pMHC-TCR avidity is bound be very low. In case of a low pMHC affinity and a high pMHC-TCR affinity, the smaller number of pMHC molecules shall still drive the overall avidity to be biased toward the lower end. The same two scenarios are now considered for the high pMHC affinity peptides. If the pMHC-TCR affinity is low, it will drive the pMHC-TCR avidity obviously to the lower end. In case of a high pMHC affinity and a high pMHC-TCR affinity, the overall avidity shall be high. It is posited that, for the small proportion of the neoepitopes that mediate tumor control in vivo and have a high affinity for MHC I (see Table 1, and some in Table 2), the pMHC-TCR affinity would, of necessity, be low, so that the pMHC-TCR avidity is also low: as recently observed (121–124), only CD8^+^ T cells with low to intermediate avidity, but not those with high avidity are able to mediate tumor control.

There is another, independent reason why the low-avidity repertoire must play an essential role in activity of neoepitope in vivo. Brennick and George et al. (62) as well as Ebrahimi-Nik et al. (31) observed that the TILs of mice immunized with low-affinity neoepitopes express significantly lower levels of markers of exhaustion (such as CD38 and LAG3 as well as 2B4 and TIGIT in combination with other markers) and significantly higher levels of stem cell markers (such as TCF1) than the TILs of mice immunized with high-affinity neoepitopes. As discussed above and shown in Figure 4B, low-affinity neoepitopes have a higher likelihood of eliciting low-avidity pMHC-TCR interactions. In conditions of chronic antigen stimulation such as a tumor, low-avidity pMHC-TCR interactions are also less likely to lead to exhausted T cells. Our recent observation that only the low-avidity (and not the high avidity), neoepitope-specific T cells mediate antitumor control in vivo supports this hypothesis (122). It is also consistent with the earlier demonstration that the pMHC-TCR affinity threshold for tumor cell killing in vivo is quite low and that a higher affinity does not add additional value (125). Using transgenic T cells against altered peptide ligands of the same model antigen in a mouse model, Shakiba et al. (121) observed previously that low-avidity CD8 responses are more effective at eradicating tumors.

Parenthetically, a lower affinity of a peptide for MHC would also reduce the total number of cells that present the peptide (compared with a high-affinity peptide). This would mean not just a decreased signal strength during each stimulation, but less frequent stimulations through the TCR, which has been shown to lead to a less exhausted phenotype (i.e., less PD1 expression and better cytokine production, refs. 126, 127). The connection between lower pMHC I affinity, lower avidity, and less exhaustion of T cells is the central pillar of the proposed simplicity on the other side of complexity.

The above hypothesis resolves the apparent paradoxes in the biology of neoepitopes. First, it explains how neoepitopes with a high or low affinity to MHC may be effective against cancers (because the pMHC affinity is less relevant than a low pMHC-TCR avidity, although a lower pMHC I affinity does bias the TCR repertoire toward the more desirable lower pMHC-TCR avidity). Second, it explains why criteria of pMHC interaction for neoepitope peptides are different from the corresponding criteria for viral and model antigens (because the T cell response against viral antigens is not circumscribed by similarity of viral antigens to self-antigens, while that of neoepitopes, is). Third, and finally, it explains why CD8 response measured in vitro is not predictive of antitumor activity in vivo (because the TCR repertoire against neoepitopes is highly sculpted and although sufficient to elicit tumor immunity in vivo, it is not of a magnitude easily measurable in vitro.).

All components of this hypothesis are fully testable. For example, methods that can modulate TCR avidity in vivo can be imagined and tested. Using such methods, neoepitopes that elicit T cells with varying avidities (regardless of pMHC I affinity) can be tested for their ability to elicit tumor control in vivo. Since regulatory T cells play a critical role in modulating the magnitude of the low-avidity CD8^+^ T cells (128), the role of agents that inhibit regulatory T cells in the antitumor activity of neoepitopes can be quantified. The degree to which the TCR repertoire against a neoepitope has been sculpted (or blunted) can be quantitatively measured; we may then explore correlations between the activity of a neoepitope in vivo and the degree to which its TCR repertoire is curtailed. The roles of precursor frequency of T cell response against a neoepitope, or the abundance of a neoepitope in the antitumor activity, can be formally determined, rather than dogmatically assumed. Measurement of T cell activation shall be better served by development of sensitive assays well beyond measurement of IFN-γ or TNF-α by flow cytometry or ELISPOT analyses; we now know enough about T cell activation and the multiple pathways and steps in such pathways to develop better assays that reflect CD8^+^ T cell activity in vivo. Advancement of understanding of the mechanisms of action (and inaction) of neoepitopes will require a series of such focused mechanistic interrogations.

## Clinical implications

### Cancer immunotherapy.

The initial clinical pursuit of neoepitope vaccines was arguably driven more by the irrational exuberance of investment capital than the strength of the scientific understanding of what neoepitopes are and how they work. That understanding is now deepening. Further clinical development of neoepitopes requires small clinical studies to proceed in tandem with the principles of immunogenicity and tumor control in vivo as they emerge from new mechanistic studies in mouse models, as discussed in the previous section. Some overriding principles may be suggested. Safety, feasibility, and immunogenicity are of course the mainstays of any phase I study; that said, neoepitope vaccine trials have never failed on these parameters. An early indication of clinical activity is desirable. Since neoepitope vaccines are almost always delivered in combination with other treatments, it might be attractive to follow the unorthodox approach where even early studies are randomized to treatment arms with and without neoepitopes. In light of the central role of regulation of self-reactivity in immune response to neoepitope, the use of reagents that inhibit the activity of regulatory T cells may be incorporated in early trials. Dogmas of predicting active neoepitopes simply based on affinity of peptides to MHC, or CD8 responses measured in vitro or ex vivo, are best discarded (while fully acknowledging their enormous impact in understanding antiviral responses). Intense monitoring of T cell immune responses, well beyond measurement of IFN-γ or TNF-α by flow cytometry or ELISPOT, will be essential to uncover true surrogates of clinical activity. At present, no such surrogates exist. More clinical trials that establish safety, feasibility, and immunogenicity of more neoepitopes will be unilluminating.

### Implications for T cell response in chronic infections and autoimmunity.

The observation that epitopes with poor affinities to MHC I can be presented by MHC and can elicit consequential, less-exhausted effector responses (26, 31, 36, 62, 77) has implications for chronic infections as well as autoimmunity. It is posited here that the T cell responses to high-affinity epitopes represent the tips of the icebergs in the landscape of T cell responses: a closer scrutiny may reveal a much broader vista of T cell responses to low-affinity epitopes (129–131), which may be particularly relevant in responses to chronic antigen exposure (Figure 4C).

## Figures and Tables

**Table 2:**
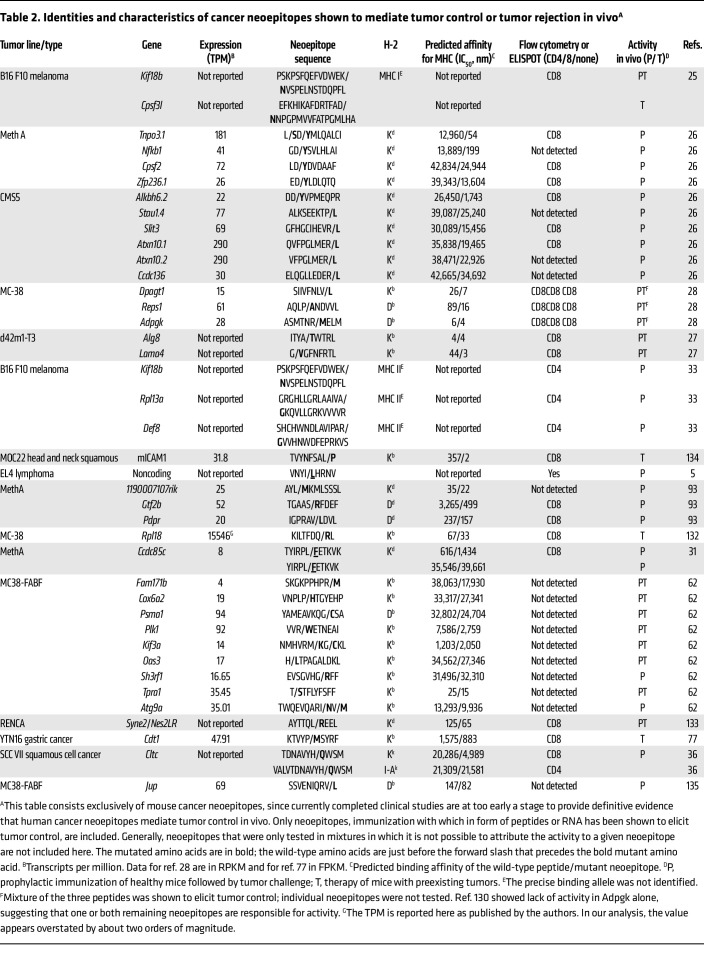
Identities and characteristics of cancer neoepitopes shown to mediate tumor control or tumor rejection in vivo^A^

**Table 1:**
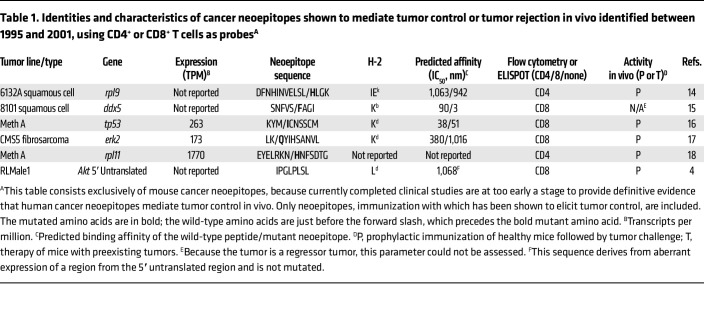
Identities and characteristics of cancer neoepitopes shown to mediate tumor control or tumor rejection in vivo identified between 1995 and 2001, using CD4^+^ or CD8^+^ T cells as probes^A^

## Version 1 03/01/2024

Electronic publication
